# Liquid Biopsy and the Translational Bridge from the TIME to the Clinic

**DOI:** 10.3390/cells11193114

**Published:** 2022-10-03

**Authors:** Paul Walker

**Affiliations:** Rody School of Medicine at East Carolina University, Greenville, NC 27834, USA; walkerp@ecu.edu

**Keywords:** liquid biopsy, tumor immune microenvironment

## Abstract

Research and advancing understanding of the tumor immune microenvironment (TIME) is vital to optimize and direct more effective cancer immune therapy. Pre-clinical bench research is vital to better understand the genomic interplay of the TIME and immune therapy responsiveness. However, a vital key to effective translational cancer research is having a bridge of translation to bring that understanding from the bench to the bedside. Without that bridge, research into the TIME will lack an efficient and effective translation into the clinic and cancer treatment decision making. As a clinical oncologist, the purpose of this commentary is to emphasize the importance of researching and improving clinical utility of the bridge, as well as the TIME research itself.

Tumor cell genomics closely intertwine with and direct the TIME compartment’s infiltration and balance of stimulatory and inhibitor immune cells, as well as non-immune stromal components. This sets up an immune ‘hot’ TIME responsive to immune therapy or an immune ‘cold’ TIME not responsive to immune therapy. The TIME is also not a static state. It is a dynamic process changing over time and can be different at different metastatic sites due to progressing metastatic clonal evolution [1].

Tissue biopsies are the standard to assess and understand the TIME. However, assessing the TIME in the clinic can be a far more difficult proposition. Repeat invasive tumor tissue biopsies carry a high procedural cost for tissue acquisition, are fraught with potential complications, cause delays in treatment, and are often not logistically practical to integrate into clinical cancer treatment decision making [2]. Unless evolving knowledge of the TIME can be translated into the clinic, that knowledge will have limited impact identifying effective (and avoiding ineffective) cancer immune treatment as well as missing potential modulation of the TIME to enhance immune therapy benefit.

## 1. Molecular Tumor Biology Can Reflect the TIME

Cancer is a disease of genomic derangements and instability driving the cancer tumor biology. The underlying molecular tumor biology with targetable driver mutations and fusions as well as actionable tumor co-mutations can reflect differing TIME and immune therapy effectiveness [3,4]. The TIME of EGFR mutation and ALK fusion NSCLC notably lacks CD8 infiltration limiting immune checkpoint blockade benefit [5]. STK11 mutations have strikingly different TIME effects based upon what co-mutations are present. STK11 mutant NSCLC with KRAS co-mutations are associated with increased IL-6, IL-1β, and CXCL7 levels along with neutrophil infiltration, yet decreased T-cell infiltration and function, decreased PD-L1 expression, and decreased stimulator of interferon genes (STING) pathway activation. Studies also point towards associated low intratumoral pH, metabolic restriction, and altered angiogenesis, all leading towards a poor immune therapy benefit [6]. However, the same STK11 mutations with associated TP53 co-mutations but without KRAS mutations, demonstrate increased STING activation and better immune therapy benefit [7]. KRAS mutant NSCLC with TP53 co-mutations also have a completely different TIME with increased IFNγ, PD-L1 expression, and increased T-cell infiltration, supporting an immune therapy benefit [6]. PD-L1 protein, mRNA, and gene amplification are all predictive of ICI responsiveness [8,9,10]. Other mutations such as POLE/POLD1 are associated with a hypermutated state reflecting a favorable immune responsive TIME as do BRCA, SMARCA4, ARID1A, BAP1, and SETD2 mutations [11,12,13,14,15,16]. Whereas STK11, KEAP1, PTEN, β-2 microglobulin mutations, MDM2 amplification, β-catenin pathway alterations, JAK1/2 loss, and oncogenic fusions are often immune therapy resistant [16,17]. Immune therapy hyperprogression in advanced NSCLC has also been associated with STK11 mutations [18]. Even differing EGFR and ERBB2 exon mutations can demonstrate differing immune therapy benefit [19].

Certain other genomic tumor biologies impact the TIME. Microsatellite instability high (MSI-H) has a unique tumor biology and TIME with increased immune cell infiltration, increased neoantigens, increased immune checkpoint expression, increased VEGFR secretion, enhanced STING activation, interferon secretion, and T cell priming, all leading to remarkable immune checkpoint inhibitor (ICI) responsiveness across tissue-site agnostic solid tumors [20,21,22]. A high neoantigen tumor mutational burden (TMB) is also associated with increased ICI responsiveness, albeit to a lesser degree [23].

## 2. Liquid Biopsy Reflects the Molecular Tumor Biology

Liquid biopsy with plasma next generation sequencing (NGS) is an evolving technology that can identify targetable and actionable molecular tumor biology from circulating tumor DNA (ctDNA) for somatic mutations and RNA (ctRNA) fusions shed from the tumor into the blood [24]. That approach has been highly effective in implementing precision oncology into the clinic and cancer treatment decision making. Although tissue and plasma molecular profiling remain complementary, cancer medicine has entered into a ‘liquid biopsy’ era where simple blood tests are beginning to efficiently identify the underlying tumor molecular biology that can effectively guide treatment.

Tissue molecular testing is fraught with tissue acquisition and spatial heterogeneity limitations. Tissue quantity is insufficient for full molecular testing in nearly half of metastatic non-small lung cancer (NSCLC) cases [25]. Even when available, tissue molecular testing only provides a tumor biology assessment limited to the site sampled and just at that one static point in time. Studies characterizing the clinical utility of liquid biopsy testing, paradoxically show that plasma NGS testing is better than tissue NGS testing for molecular testing. In separate studies with parallel plasma and tissue NGS testing in advanced NSCLC, tissue missed 33–43% of the mutations identified with the complementary testing whereas plasma identified 80–87% of those mutations [26,27]. Plasma NGS testing can also overcome the limiting tissue heterogeneity identifying resistant clones and providing a broader assessment of the evolving tumor biology than a single biopsy site [28]. Most importantly, treatment guided by plasma NGS molecular results have better survival outcomes than treatment based upon tissue molecular testing [29]. This has led the International Association for the Study of Lung Cancer to advocate a ‘plasma first’ molecular testing approach in NSCLC [30]. Liquid biopsies with plasma NGS testing have been highly effective in assessing and reflecting the molecular tumor biology.

However, liquid biopsies are also fraught with limitations. Shedding of ctDNA/RNA from the tumor microenvironment by apoptosis and tumor necrosis is tumor burden, tumor compartment, and tumor genomic microenvironment dependent. The greater the tumor burden and stage, the greater the ctDNA/RNA shedding. In stage I NSCLC 45% will have detectable ctDNA shedding, increasing to 72–75% in lymph node positive stages II/III, and 83% in stage IV [31]. This is typical distribution across a variety of cancers. Non-shedders of ctDNA/RNA have a less aggressive tumor biology with a more favorable prognosis irrespective of stage compared to shedders [32]. In a study of advanced NSCLC patients, multivariate analysis identified visceral metastases, tumor burden, EGFR mutations, and TP53 mutations as independent predictors of increased ctDNA shedding [33]. Higher pathologic stage, nodal metastases, solid adenocarcinoma histology, tumor necrosis, and frequent mitosis were associated with higher ctDNA shedding in resectable NSCLC [34].

Plasma NGS testing can identify ctDNA/RNA alterations, MSI, and TMB associated with ICI sensitive and resistant molecular tumor biologies. Serial ctDNA can also be used to monitor ICI responsiveness with decreasing variant allele fractions and/or clearance predicting durable cancer survival and conversely, progressing disease with increasing ctDNA/RNA [35,36].

## 3. A Composite Assay Liquid Biopsy Is Needed to Fully Reflect the TIME

However, the TIME is a far more complex entity and is more than mutational tumor biology. ICI responsive immune hot tumors are infiltrated with leukocytes and tumor specific CD8 T-cells. Intratumoral chemokines, IFN-gamma, PD-L1 and, indole 2,3-dioxygenase (IDO) are part of the immune hot TIME responding to ICI therapies. Conversely ICI resistant immune cold tumors express TGF-beta and tumor associated macrophages [37]. Other immune checkpoints such as LAG-3 and TIGIT, and others, may need to be targeted [38,39]. Additionally, the gut microbiome, spatial TIME CD8 T-cell infiltration and tumor cell contact, myeloid inhibition of CD8 T-cells, immunosuppressive myeloid derived suppressor cells, inhibitory T-Reg cells, activated dendritic cells, intratumoral hypoxia, B-cells, cancer associated fibroblasts, all play an important aspect of the TIME [40]. Tumor infiltrating lymphocytes genomic signatures have been shown to be pan-cancer prognostic and predictive of immune therapy benefit [41]. Host inflammatory markers, as simple as a neutrophil-lymphocyte ratio, C-reactive protein levels, and albumin levels, as well as more complex proteomic signatures impact ICI therapy outcomes [42,43]. A composite liquid biopsy of PD-L1with other immune checkpoints, MSI, TMB, ICI sensitive and resistance ctDNA/RNA alterations, immune cellular levels, and incorporating these other TIME, as well as host parameters, is well likely needed to best reflect the TIME and predict ICI treatment benefit.

## 4. Improving Liquid Biopsy to Better Assess the TIME

The optimal make-up and balance of a composite TIME/ICI predictive liquid biopsy assay is one important research focus. Another aspect of clinically impactful liquid biopsy research is increasing the release of tumor DNA/RNA and other TIME markers to better reflect the TIME. Lack of DNA/RNA shedding, and potential undetectable sub-clones, can limit liquid biopsy assessment of the TIME. However, it is known that certain types of tumor site tissue disruption can increase ctDNA/RNA release. This would further enhance the clinical utility of liquid biopsies in assessing the tumor biology and guiding more effective cancer treatment.

Therapeutic TIME disruption has been shown to enhance ICI effectiveness. Oncolytic virus directly disrupts the TIME [44]. Nanosecond pulsed electric field (PEF) energy delivery has been shown to inhibit tumor growth locally and alter the intratumoral immune cell infiltration and response [45]. Stereotactic radiosurgery (SRS) will disrupt the TIME inducing tumor-specific CD8 T-cells and other TIME changes [46,47].

SRS has been shown to increase release of ctDNA even from early-stage cancers. SRS causes a marked rise in ctDNA at the 24 h mark after the first fraction. In a study of fifteen patients with stage I NSCLC treated by SRS, only 47% had identified ctDNA prior to treatment. However, repeat testing within 24 h of the first SRS fraction showed a median 4.5-fold increase in ctDNA [48]. ctDNA was also increased from baseline upon completion of SRS. Higher levels of ctDNA were noted at 24 h after irradiation than in pre-irradiation samples with a peak at 7 days [49]. There was also a notable increase in targetable ctDNA EGFR mutant allele fractions [50]. One of these therapeutic TIME disruption technologies could certainly be used to enhance ctDNA/RNA and other TIME parameters release into the circulation for liquid biopsy detection.

Whether a lesser physical disruption of the TIME with a simple tissue biopsy achieve this same increase ctDNA/RNA release is not well studied. Nor is there a known optimal time of drawing blood for plasma NGS testing relative to a tissue biopsy to obtain a maximal ctDNA/RNA yield for diagnostic and therapeutic information.

Multiple prostate tissue biopsies have been shown to increase ctDNA release at 60–120 min post-biopsy. In a cohort of thirty-eight patients undergoing and cfDNA testing pre-biopsy and 10, 30, 60, and 120 min post-biopsy, cfDNA peaked at 60–120 min. At 60 min, pre-biopsy median 2.76 ng/mL to 3.62 ng/mL (*p* = 0.0023) and at 120 min, pre-biopsy 5.1 ng/mL to 7.05 ng/mL (*p* = 0.0023). Patient-specific somatic mutations were compared pre-biopsy and post-biopsy. The number of reads at the patient-specific mutations increased from 3.9 to 164 times the ratio amounts present circulating before the biopsy. This supports the post-biopsy plasma was enriched with specific ctDNA. The increase was felt to be due to direct physical damage and disruption with cellular breaks and necrosis [51].

Tumor tissue disruption does increase the shedding of ctDNA/RNA from baseline pre-tissue disruption levels. Incorporating these therapeutic tumor disruption modalities could have a dual role of local cancer treatment and enhancing the liquid biopsy identification of the TIME and immune therapy benefit.

## 5. Improving the Bridge to Translate the TIME into Effective Therapy

Translating the TIME advances from the laboratory bench into the clinic bedside is vital to improving cancer outcomes for patients. The purpose of this commentary was to emphasize the perspective and importance of researching into improving the translational bridge itself and not just focusing on research of the TIME. The quick turn-around times, broadening molecular tumor biology findings, and the extending clinical utility of plasma NGS testing has ushered in the ‘liquid biopsy’ era of cancer medicine. A research focus of this translational bridge is important to improve the translation of the advances and understanding of the TIME into the clinic. Expanded correlation of the genomic tumor biology of the TIME with plasma NGS findings is important. Additional research into expanding the clinical utility development of a composite TIME/ICI responsive liquid biopsy by incorporating other TIME as well as host parameters is needed. Something as simple as research into maximizing the release of ctDNA/RNA and other TIME biomarkers could be important to improve the clinically needed translational bridge. Determining an optimal timing of a liquid biopsy blood draw relative to tissue disruption could also have a significant impact in achieving improved TIME yields.

Research to improve the bridge can be as important as the knowledge transported on it. Without a strong bridge from the bench to the bedside, translating cancer research will not achieve what we all strive for…advancing and optimizing effective cancer immune therapies.

## Data Availability

Not applicable.
